# Pulmonary arterial hypertension related to congenital heart disease with a left-to-right shunt: phenotypic spectrum and approach to management

**DOI:** 10.3389/fcvm.2024.1360555

**Published:** 2024-05-09

**Authors:** Paolo Ferrero, Andrew Constantine, Massimo Chessa, Konstantinos Dimopoulos

**Affiliations:** ^1^ ACHD Unit, IRCCS-Policlinico San Donato, Milan, Italy; ^2^ Adult Congenital Heart Disease Unit, Queen Elizabeth Hospital, Birmingham, United Kingdom; ^3^ Vita Salute San Raffaele University, Milan, Italy; ^4^ Adult Congenital Heart Centre and Centre for Pulmonary Hypertension, Royal Brompton Hospital, London, United Kingdom; ^5^ National Heart and Lung Institute, Imperial College London, London, United Kingdom

**Keywords:** pulmonary hypertension (PAH), congenital heart disease, left-right shunt, pathophysiology, management

## Abstract

Patients with pulmonary hypertension associated with a left-right shunt include a wide spectrum of pathophysiological substrates, ranging from those characterized by pulmonary over-circulation to those with advanced pulmonary vascular disease. The former group may benefit from shunt repair in carefully selected cases but, when advanced pulmonary vascular disease has developed, defect closure should be avoided, and pulmonary vasodilators may be used to improve effort tolerance and hemodynamics. There is a paucity of evidence, however, to support decision-making in the care of these patients. We discuss the principles of management in patients with pulmonary hypertension and a predominant left-right shunt. The recommendations and statements made in this paper are based on pathophysiological considerations and expert opinion.

## 1 Introduction

Data from clinical trials have clearly demonstrated the long-term efficacy of pulmonary arterial hypertension (PAH) therapies in certain cohorts of patients with PAH related to congenital heart disease (CHD) (1). However, it has to be underscored that PAH related to CHD (PAH-CHD) represents a very heterogeneous population, with major differences in terms of cardiovascular pathophysiology and prognosis between the 4 major subgroups: Eisenmenger syndrome, PAH and left-right shunt, PAH with a small/coincidental shunt, and PAH after shunt CHD repair (2, 3).

There is, nowadays, sufficient evidence to support the use of PAH therapies in patients with Eisenmenger syndrome, resulting in an improvement in effort tolerance, functional class and pulmonary hemodynamics (4–7). PAH therapies are also routinely used in patients with evidence of pulmonary vascular disease (PVD) after shunt repair. Indeed, pulmonary hypertension is detected in up to 5%–10% of repaired patients (8), who often display a clinical phenotype similar to idiopathic PAH in terms of prognosis and therapeutic response (3, 9). In both cohorts, a drop in pulmonary vascular resistance (PVR) and increase in pulmonary blood flow at rest, and especially on exertion, reduces the afterload to the right ventricle (RV) and optimizes oxygen delivery to the peripheral tissues.

The potential benefit of PAH therapies for patients with mild or moderate PAH associated with a left-right shunt is less clear, and evidence is lacking. Patients with milder forms of PAH may undergo repair while, for the remainder, there is little evidence to guide management. Despite the paucity of data, a significant proportion of these patients do receive PAH therapies in specialist centers (10). The aim of this review is to appraise the unique and dynamic pathophysiology of PAH associated with a left-right shunt and discuss practical management modalities in this important subset of CHD patients.

## 2 Epidemiology

Although the incidence of PAH-CHD is declining in developed countries, it is still prevalent globally, ranging from 4% to 28% of adults with CHD (9, 10). An accurate estimation of the prevalence of PAH-CHD with a left-right shunt is particularly challenging, as few reports focus on this population (11).

In the CONCOR CHD registry, 15 out of 135 PAH-CHD patients had a left-right shunt, a minority of whom were on PAH therapies (8). In another large multicenter PAH-CHD registry, a quarter of patients were non-Eisenmenger (167/680, 24%), and all were on targeted therapy (9). These numbers, however, include patients from the 3 non-Eisenmenger categories (left-right shunt, coincidental shunt and post-repair), and should be interpreted with caution. Indeed, patients with PAH and left-right shunts represent a distinct entity, with little evidence in terms of prevalence and management, hence they are treated empirically in clinical practice. It is also important to remember that many such patients may, over time, progress to Eisenmenger syndrome or move into the post-repair group if they undergo percutaneous or surgical repair and are found to have residual PAH.

## 3 Pathophysiology

Since Paul Wood's treatise on the pathophysiology of Eisenmenger syndrome in the 1950's (12), many hypotheses have been advanced on how PVD develops in patients with congenital shunts. It is now accepted that PVD is a continuum of anatomic and pathophysiological changes, which may eventually lead to irreversible disease depending on the nature and severity of the shunt. Several genetic, physiological and anatomic variables may modulate the progression towards advanced PVD, with a window of reparability of the defect that can vary significantly between individuals (13). There are also small cohorts of young children in whom the pulmonary circulation does not remodel appropriately after birth and histological changes in the lung, such as intimal proliferation, occur too early in life to be significantly influenced by cumulative shear stress on the pulmonary vasculature (14).

Patients who are deemed non-reparable due to established PVD are often considered for PAH therapies, though the rationale for the use of pulmonary vasodilators in the presence of a sizable left-right shunt has been debated for some time (15). Moreover, the clinical and hemodynamic targets of PAH therapy in this population often differ to the other subsets of PAH-CHD.

### 3.1 Genetic modulators

The role of genetic factors in modulating the onset and disease severity of idiopathic PAH is nowadays established, with mutations in the Bone Morphogenetic Protein Receptor II gene (*BMPR2*) being the most common (16). More recently, a role for pathogenetic polymorphisms in PAH-CHD has been suggested: *BMPR2* mutations were identified in 6% of adults and children with PAH-CHD (mainly Eisenmenger syndrome or post-repair), although the role of *BMPR2* in PAH-CHD remains unclear (17). An increased prevalence of mutations in the SRY-Box Transcription Factor 17 gene (*SOX-17*) and T-box transcription factor gene (*TBX4*) has been identified in patients who developed PAH after ventricular septal defect repair and patients with pre-tricuspid shunts and Eisenmenger physiology (18–20). Other genes (*ABCC8* and *SMAD1*) have been described in patients with PAH associated with a small/coincidental defect (18). Genetic factors may explain the variability in the development of PVD in patients with pre-tricuspid shunts, in whom the increased pulmonary blood flow alone is deemed insufficient for the development of PVD (21).

The molecular mechanisms influencing the onset and progression of PAH have been further dissected, suggesting that epigenetic factors play a major role in shaping the clinical expression of the different genetic variants. These factors, which include sequences of non-coding RNA, micro-RNA and protein polymorphisms, are still poorly understood and a comprehensive appraisal of this topic is beyond the scope of this review (22). Recent mechanistic work has shown a link between signals regulating cellular proliferation and differentiation, and the biology of pulmonary smooth muscle cells (23). Different levels of expression of proteins involved in smooth muscle cell proliferation correlate with the severity of PVD in animal models of flow-associated PAH; such proteins may have a protective role against vascular remodeling in the presence of a left-right shunt (23). These data shed new light on the pathogenesis of PAH-CHD, moving away from the traditional view that this is a condition triggered exclusively by hemodynamics. Genetics are likely to play a role on whether PVD develops (especially in patients with pre-tricuspid shunts), and how rapidly it progresses, influencing operability, the response to PAH therapies, and prognosis in this cohort.

### 3.2 Hemodynamic modulators

Shunts occurring at different anatomic levels portend different risks of PVD. Large post-tricuspid shunts result in pressure and volume load to the pulmonary circulation and are much more likely to cause PVD than pre-tricuspid shunts (i.e., atrial septal defects). This is particularly true for congenital shunts between the great arteries, such as common arterial trunk, aortopulmonary window or large patent ductus arteriosus, which generate significant shear stress to the pulmonary circulation throughout the cardiac cycle. If left unrepaired, they are likely to cause severe PVD and evolve to Eisenmenger physiology early in life (12). Wagenvoort et al. demonstrated that pulmonary artery banding in patients with post-tricuspid shunts and histological evidence of PVD did reverse the disease in some, most likely those with milder forms of PVD, demonstrating the spectrum of disease severity and difficulties in establishing who has developed irreversible pulmonary arteriopathy by means of cardiac catheterization or even lung biopsy (24, 25).

**Figure 1:**
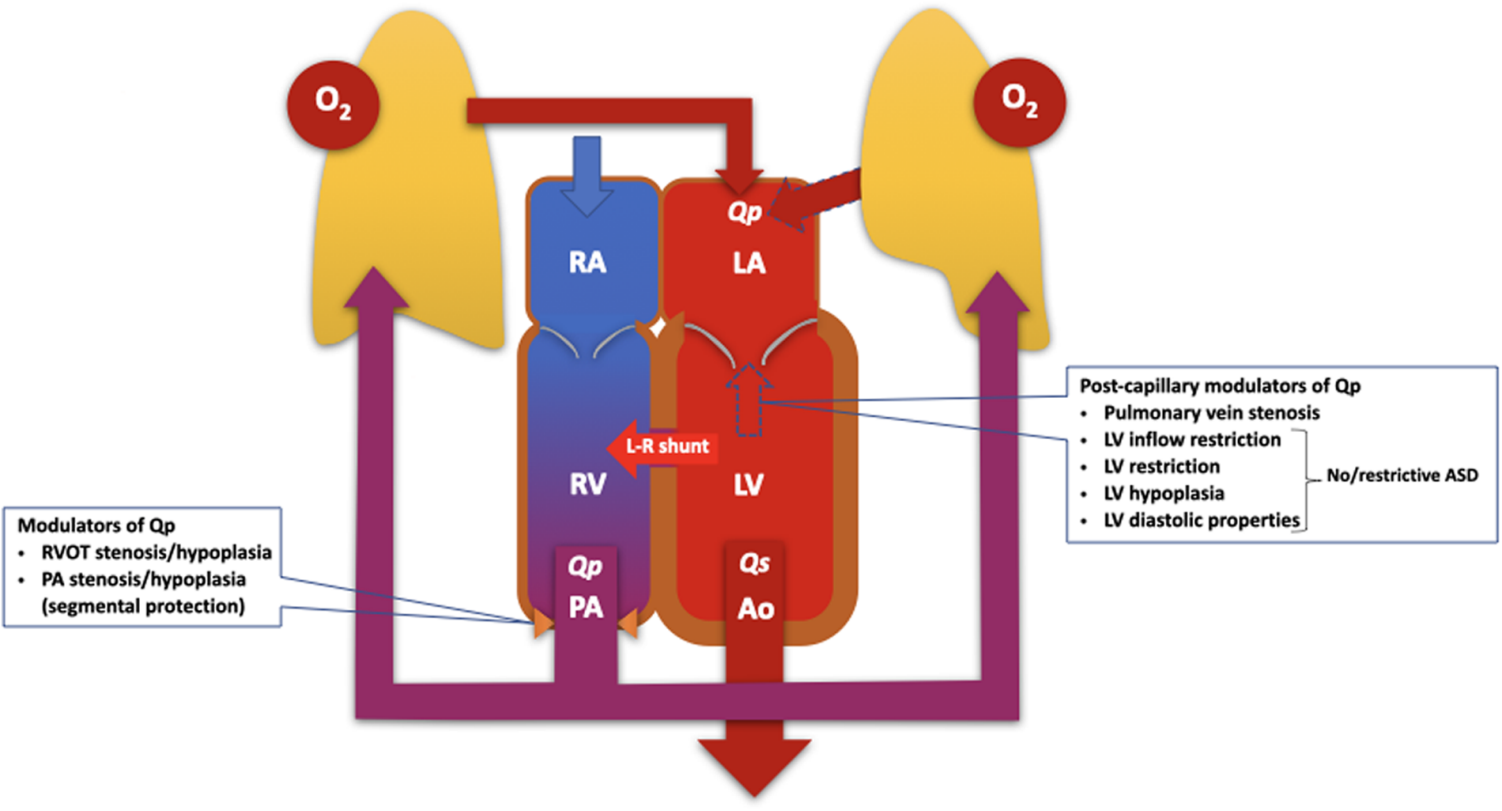
Diagram illustrating the pathophysiology of the pulmonary and systemic circulations, which work in parallel to modulate pulmonary blood flow and the severity of the left-right (L-R) shunt. The modulators of pulmonary blood flow and shunt traction can, in principle, be classified as pre- and post-capillary, and should be considered when assessing patients with PAH and left-right shunts. ASD, atrial septal defect; LA, left atrium; LV, left ventricle/ventricular; O_2_, oxygen; PA, pulmonary artery; Qp, pulmonary blood flow; Qs, systemic blood flow; RA, right atrium; RV, right ventricle; RVOT, right ventricular outflow tract.

When assessing pulmonary blood flow and shunt fraction, several factors need to be taken into consideration. For pre-tricuspid shunts, the shunt fraction depends not only on the size of the defect, but also on the pressure gradient between the atria. Conditions that increase left atrial pressure, such as left-sided valve disease, systemic ventricular dysfunction, are likely to promote left-right shunting, while conditions that increase right atrial pressure such as tricuspid valve disease, right ventricular outflow tract obstruction or pulmonary hypertension, reduce left-right shunting and may cause shunt reversal (Figure 1).

The pathophysiology becomes even more complicated in the presence of segmental pulmonary hypertension, i.e., when PVD develops in some but not all lung segments (26). This is typical of patients with tetralogy of Fallot with pulmonary atresia, but can also occur in patients with a large post-tricuspid shunt and branch pulmonary artery stenosis.

Occasionally, patients with large, non-restrictive post-tricuspid communications present late with surprisingly mild PAH and a predominant left-right shunt, with or without signs of pulmonary congestion. In the absence of right ventricular outflow tract obstruction or branch pulmonary artery stenosis that may protect the pulmonary vascular bed, such patients are deemed to have a “compliant” pulmonary circulation that is less sensitive to shear stress and the overload caused by the shunt. Whether or not this phenotype is genetically driven remains to be determined.

## 4 Management of PAH associated with left-right shunts

There is very little evidence on the use of PAH therapies in patients with CHD and a predominant left-right shunt (1, 27–31). In other PAH cohorts, these therapies are used to lower PVR and increase pulmonary blood flow at rest and on effort, reducing RV afterload and improving the coupling between the RV and the pulmonary circulation. In patients with mild PAH and a sizable left-right shunt (i.e., ratio of pulmonary-to-systemic blood flow or Qp/Qs > 1.5), PAH therapies are likely to further increase the already excessive pulmonary blood flow, potentially exacerbating the overload to the right or left ventricle and the pulmonary circulation. In such cases, PAH therapy only makes sense as a means of reducing PVR to levels that would allow defect repair (the so-called “treat-and-repair” approach, first proposed by Dimopoulos et al.) (32). The use of triple-combination therapy, a cornerstone in the management of severe idiopathic PAH, lacks evidence in this patient population. Patients with predominant left-right shunts are often excluded or underrepresented in PAH-CHD cohorts studying the impact of combination therapy (33, 34). Cardiac catheterization is required prior to and after establishing PAH therapies to document the decrease in PVR to levels that would allow partial (or complete) closure of the defect, thus abolishing ventricular overload and reducing the risk of progression of PVD. Current ESC adult CHD guidelines make clear recommendations on a treat-and-repair strategy for patients with an atrial septal defect and baseline PVR ≥ 5 WU (35). They recommend fenestrated closure of the defect after PAH therapy if the PVR drops to <5 WU and there is evidence of RV volume overload with a Qp/Qs > 1.5. It is worth noting that this cut-off is reasonably conservative; in the past, atrial septal defects were closed with a PVR of 6–8 WU (36). Fenestrated closure of pre-tricuspid shunts in patients with with PAH, with concurrent initiation of PAH therapy at the time of repair has been described, though this is not endorsed in the same way as “treat-and-repair” in the ESC ACHD guidelines (35, 37). Indeed, a significant drop in PVR after therapy is deemed essential for considering defect closure, and there is a risk that patients who do not adequately respond to PAH therapies long-term may be negatively affected by even partial closure of the defect. It is generally expected that PAH therapies should be prescribed indefinitely in patients undergoing successful treat-and-repair (who now belong to the post-repair PAH-CHD subgroup), though cardiac catheterization and close monitoring, with appropriate adjustment of therapies, is recommended post-repair.

With regards to post-tricuspid, left-right shunts with a PVR ≥ 5 WU, there is very little evidence to guide management and guidelines recommend referral to expert centers, who may consider a similar treat-and-repair approach. Expert centers may choose to initiate and escalate PAH therapies (as single or combination therapy) in symptomatic patients, monitoring hemodynamics for a significant drop in PVR and/or significant increase in the left-right shunt and ventricular overload. There are several case reports and series describing patients with post-tricuspid shunts and established PVD who responded to PAH therapy and underwent repair of the defect (38, 39). However, patients with post-tricuspid shunts often present with more severe PVD, with a higher baseline PVR than their counterparts with pre-tricuspid shunts, making a treat-and-repair strategy less attractive. Moreover, many reports of successful treat-and-repair in post-tricuspid shunts are on patients from Asia, and a more favorable genetic background cannot be excluded. Finally, the changes in hemodynamic operability criteria over time, differences in consensus between analogous guidelines and variability in management by center and region, create challenges for the use of “real-world” clinical data to infer safety (35, 40). The magnitude of PVR drop achieved with PAH therapy in post-tricuspid shunts varies significantly, between 20% and 40%, roughly reproducing the relative reduction reported in the interventional arm of clinical trials of idiopathic PAH (31, 41–43). The duration of pre-repair treatment with PAH therapies has also not been standardized (36, 38, 44). It is likely that most patients will require long-term treatment after repair, though this will depend on post-repair hemodynamics.

The attention and expertise required in the performance and interpretation of cardiac catheterization in such patients cannot be overstated. Quality control is essential and pitfalls should be avoided, e.g., in the presence of multiple sources of pulmonary blood flow (patent ductus arteriosus, aortopulmonary collaterals), the positions used to sample blood for calculation of mixed venous saturations and the choice of equation used, use of sedation or anesthesia that can influence hemodynamics, administration of large doses of oxygen that can confound shunt calculations, etc. Acute vasodilator challenge is not currently advocated in the guidelines but may provide information on the compliance of the pulmonary vasculature (1, 35).

While short-term outcomes after treat-and-repair have been promising in case reports and case series, the long-term effects of closing defects in these patients remains uncertain (4, 30). An added complication to the management of PAH-CHD patients with a left-right shunt is the lack of data on risk stratification, which forms the basis for decision-making in other PAH cohorts. Previous versions of the ESC/ERS pulmonary hypertension guidelines proposed an extended three-strata prognostic model for PAH, which should guide management but is not validated for CHD, especially patients with left-right shunts (29). This model has been refined over the years and updated prognostic models have been developed based on European and US cohorts. A simplified four-strata model that utilizes 3 clinical parameters (WHO functional class, 6-min walk distance and brain natriuretic peptide) was proposed in the latest 2022 guidelines; the two major studies supporting this model included very few non-Eisenmenger CHD patients (1, 28). This model has been applied, but not formally validated, in patients with PAH-CHD (45).

## 5 Proposal of a practical approach

When considering PAH therapies in patients PAH-CHD and left-right shunts, expert centers may ask the following 2 pivotal questions:

1. What are the aims of therapy and the clinical endpoints set for assessing efficacy?
2. How should one select patients that are most likely to benefit from PAH therapies?

The potential aims of PAH therapy in this cohort are either to:

- achieve operability criteria, i.e., modify pulmonary hemodynamics to fulfill recommended criteria for repair (as part of a treat-and-repair approach) (35), or
- produce an improvement in functional status, exercise capacity and, possibly, prognosis.

Defects that can be repaired percutaneously are more attractive for a treat-and-repair approach, as surgery requiring cardio-pulmonary bypass carries substantial risks (e.g., perioperative pulmonary hypertension crisis), even in patients on effective PAH therapy. Close perioperative monitoring is essential to minimize perioperative complications, with a low threshold for escalation of PAH therapies and hemodynamic support, when required. Repeat invasive hemodynamic assessment is required to establish post-repair hemodynamics and decide on the intensity of PAH therapy, with the aim of optimizing long-term outcomes, in a patient who is now classifiable as post-repair PAH-CHD. In this setting, the decision to withdraw one or more therapies in a patient with normal or near-normal PVR is difficult and great caution is recommended as PVD is more than likely to persist and may progress.

In patients with more severe PVD but still with predominant left-right shunting (i.e., not yet fulfilling Eisenmenger criteria), a treat-and-repair strategy is less likely to be successful; repairing a defect in a patient who requires very aggressive PAH therapy to achieve hemodynamic criteria for operability may not be wise and the benefits in terms of abolishing the left-right shunt may not outweigh the risks of the intervention and likelihood of progressive severe PVD after repair of the defect. There may, however, be merits in using pulmonary vasodilators in an attempt to improve ventriculo-vascular and gas exchange coupling, optimizing RV stroke volume and improving pulmonary blood flow through better distribution to relatively normal V/Q units (46). In this setting, PAH therapies can lower PVR and RV afterload, though it is likely that the left-right shunt and ventricular volume overload will increase. Moreover, it is unclear how this strategy may influence (accelerate or delay) the natural evolution towards Eisenmenger physiology.

**Figure 2:**
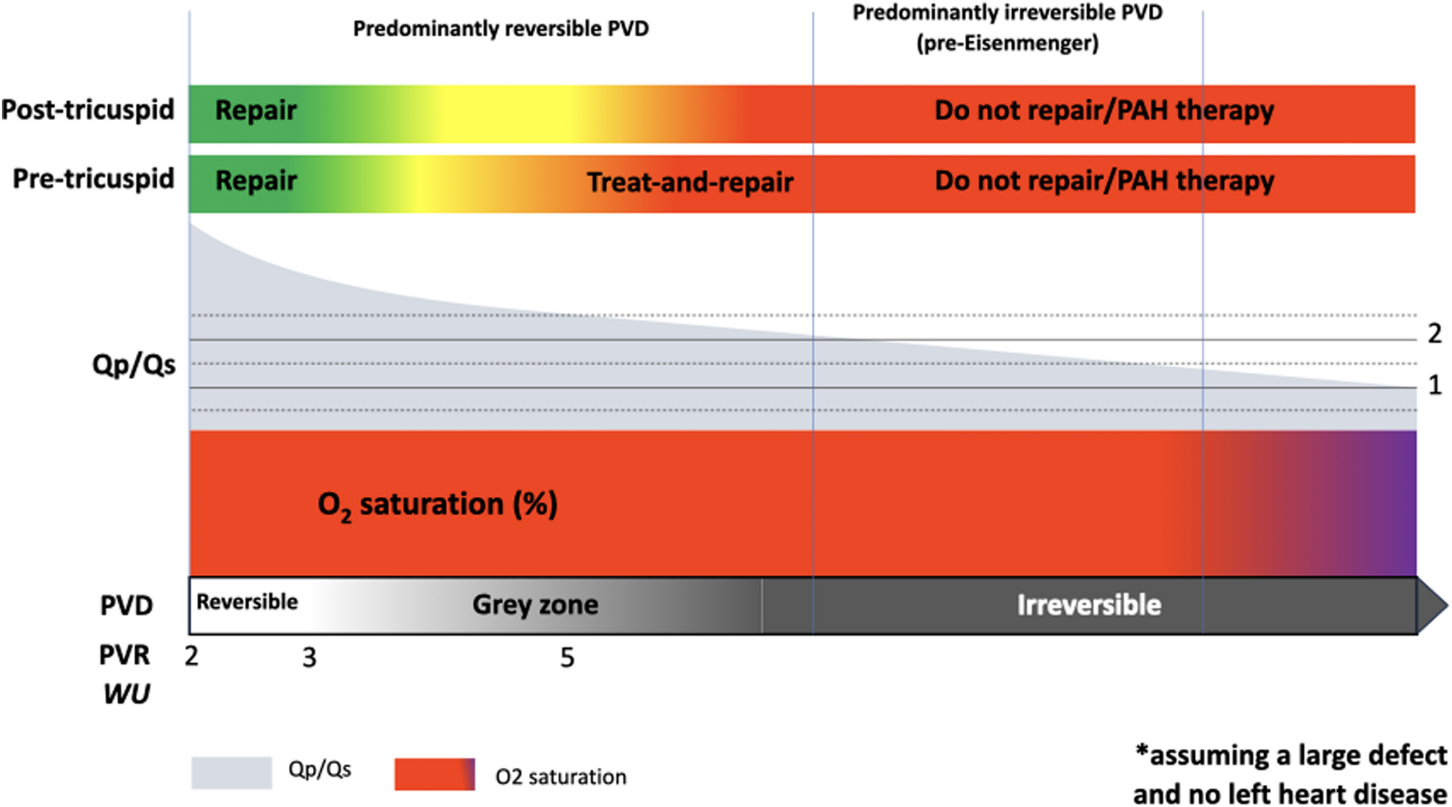
Graphical summary of PAH-CHD with a left-right shunt, spanning from the predominantly reversible end of the spectrum, with mildly elevated pulmonary vascular resistance (PVR) and high shunt fraction (Qp/Qs), to the predominantly irreversible pulmonary vascular disease (PVD) or pre-Eisenmenger end. This schematic assumes a large congenital heart defect and no significant left heart disease.

It, therefore, makes sense to categorize patients with PAH-CHD and a left-right shunt in two distinct groups, assuming a large pre- or post-tricuspid defect and no left heart disease or associated lesions (Figure 2):

- The *predominantly reversible* PVD type refers to patients with a large left-right shunt and evidence of, typically, mild pulmonary hypertension. The vast majority of these patients are deemed operable, yet some fall into a decision-making “grey zone” that requires careful expert assessment. In this group, PAH therapies may be used as part of a treat-and-repair approach, even though this is only recommended explicitly for atrial septal defects. Once repair is achieved, PAH therapies are typically continued long-term, though adjustment may be required depending on hemodynamics, immediately post-intervention and long-term. Patients in whom treat-and-repair fails in terms of an insufficient drop in PVR despite adequate therapies are likely to have a predominant irreversible component and should be classified as such (see below).
- The *predominantly irreversible* PVD or *pre-Eisenmenger* type have a left-right shunt smaller than expected for the size of the defect, but without cyanosis at rest or on exercise. In this group, PAH therapies may be considered under strict surveillance to improve hemodynamics and RV function, though evidence is lacking. The possible benefit of PAH therapies needs to be balanced against the risk of augmenting the left-right shunt and precipitating RV failure due to worsening volume overload. In these patients, PAH therapy is unlikely to result in a sufficient drop in PVR that would allow defect closure.

While patients with PAH-CHD and a prevalent left-right shunt are common, a systematic description of the spectrum of disease contained within this group requires a return to the anatomic, pathophysiological, histologic, and hemodynamic concepts of reversible vs. irreversible PVD. Unfortunately, despite decades of work, 70 years since the systematic description of Eisenmenger physiology by Wood, and almost 40 years since the pioneering work of Wagenvoort et al., the concept of reversibility eludes us (12, 25). This impacts our decision making, both in terms of operability and use of PAH therapies. Yet, there is a spectrum within PAH-CHD with a left-right shunt that can only be conceptualized fully in terms of the severity of PVD and its reversibility i.e., whether our interventions are likely to impact favorably on the natural history of this progressive, life-limiting condition. Unfortunately, the transition point from predominantly reversible to predominantly irreversible disease cannot be easily defined based on current evidence, and this is reflected in the latest clinical recommendations: ESC guidelines use <5 WU for the definition of operability and by extension reversibility, yet they recognize the significant uncertainty and variability in practice and recommend careful individual assessment in expert centers for patients with a post-tricuspid shunt and a PVR ≥ 5 WU (35). The classification of children into predominantly reversible and irreversible PVD, while clinically important, is significantly complicated by the dynamic nature of the pulmonary vasculature in early life, and its variable response to large shunts (confounded by genetic and other factors). The physiological post-natal drop in PVR is typically followed by the emergence of an overcirculating phenotype. This is characterized by heart failure, driven by the large left-right shunt and indicative of a still compliant pulmonary vascular bed. There are, however, infants in whom the expected drop in PVR after birth does not occur and, thus, never experience a window of operability (14).

As risk stratification models are not available for this cohort of patients, it makes sense to monitor standard clinical parameters, such as WHO functional class, 6-min walk distance, natriuretic peptide levels and echocardiographic indexes of RV dysfunction and coupling to the pulmonary circulation. Cardiac magnetic resonance imaging can be extremely useful for monitoring RV function and obtaining non-invasive measures of pulmonary blood flow and shunt fraction. Cardiopulmonary exercise testing (CPET), for patients who can perform this test (i.e., without severely limited exercise capacity or significant learning disability), is a safe and useful non-invasive method for assessing functional capacity. It is part of the guideline-recommended initial risk assessment of patients with PAH (1). The development of progressive oxygen desaturation on exertion may suggest that a patient is approaching Eisenmenger physiology, while improvements in oxygen saturation and exercise parameters after PAH therapy are evidence of response to treatment. Ventilatory inefficiency and ventilation-perfusion mismatching, as determined by a raised the ratio between ventilation and CO_2_ production (VE/VCO_2_ slope), is related to outcome in PAH and non-cyanotic CHD cohorts (47–49). This, along with other markers of functional capacity, may be used for risk stratification. Right heart catheterization prior to and post-initiation of therapies is instrumental in monitoring hemodynamics (e.g., shunt fraction and PVR) and identifying patients who have achieved operability criteria, or may benefit from escalation of therapy. In patients not eligible for repair, one should aim to achieve a balance between optimizing pulmonary hemodynamics, RV afterload and oxygen exchange, while avoiding a significant increase Qp/Qs that may be detrimental to RV or left ventricle (for pre- or post-tricuspid shunts, respectively) (Figure 2).

## Conclusions

The management of patients with PAH and a left-right shunt is particularly challenging, as this group includes individuals with a wide range of hemodynamics, and evidence is extremely limited. Potential indications for PAH therapies differ according to underlying anatomy and hemodynamics, with two major clinical phenotypes: *predominantly irreversible* (*pre-Eisenmenger*) vs. *predominantly reversible* type. A tailored expert approach is mandatory, with close monitoring after defect repair and/or use of PAH therapies.
